# Quantitative trait locus analysis for endophenotypes reveals genetic substrates of core symptom domains and neurocognitive function in autism spectrum disorder

**DOI:** 10.1038/s41398-022-02179-3

**Published:** 2022-09-24

**Authors:** In-Hee Lee, Ekaterina Koelliker, Sek Won Kong

**Affiliations:** 1 Computational Health Informatics Program, Boston Children’s Hospital, Boston, MA 02215 USA; 2 Psychology Department, Colby College, Waterville, ME 04901 USA; 3 Department of Pediatrics, Harvard Medical School, Boston, MA 02115 USA

**Keywords:** Autism spectrum disorders, Genomics

## Abstract

Autism spectrum disorder (ASD) represents a heterogeneous group of neurodevelopmental disorders and is largely attributable to genetic risk factors. Phenotypic and genetic heterogeneity of ASD have been well-recognized; however, genetic substrates for endophenotypes that constitute phenotypic heterogeneity are not yet known. In the present study, we compiled data from the Autism Genetic Resource Exchange, which contains the demographic and detailed phenotype information of 11,961 individuals. Notably, the whole-genome sequencing data available from MSSNG and iHART for 3833 individuals in this dataset was used to perform an endophenotype-wide association study. Using a linear mixed model, genome-wide association analyses were performed for 29 endophenotype scores and 0.58 million common variants with variant allele frequency ≥ 5%. We discovered significant associations between 9 genetic variants and 6 endophenotype scores comprising neurocognitive development and severity scores for core symptoms of ASD at a significance threshold of *p* < 5 × 10^–7^. Of note, the Stereotyped Behaviors and Restricted Interests total score in Autism Diagnostic Observation Schedule Module 3 was significantly associated with multiple variants in the *VPS13B* gene, a causal gene for Cohen syndrome and a candidate gene for syndromic ASD. Our findings yielded loci with small effect sizes due to the moderate sample size and, thus, require validation in another cohort. Nonetheless, our endophenotype-wide association analysis extends previous candidate gene discovery in the context of genotype and endophenotype association. As a result, these candidate genes may be responsible for specific traits that constitute core symptoms and neurocognitive function of ASD rather than the disorder itself.

## Introduction

Autism spectrum disorder (ASD) is a neurodevelopmental disorder characterized by deficits in verbal and nonverbal communication, and social interactions that co-occur with restricted and repetitive behaviors (RRBs). Impairments must be present in three core domains (communication, social, and behavior) for a diagnosis of ASD to be made [1]. Amongst individuals with ASD, phenotypic heterogeneity in adaptative functioning, cognitive development, and neurological comorbidities such as epilepsy, hydrocephalus, and sleep disorders is immense [2]. The Autism Diagnostic Interview-Revised (ADI-R) and the Autism Diagnostic Observation Schedule (ADOS) are widely regarded as the “gold standards” for ASD diagnosis as they represent criteria from the Diagnostic and Statistical Manual of Mental Disorders—5^th^ edition (DSM-5) [3]. In the realm of cognitive functioning, intellectual disability (intelligence quotient (IQ) ≤ 70) affects 33% of individuals with ASD [4]. As a result, the assessment of cognitive and adaptive abilities extending beyond the traditional triad of symptoms is useful for assessing ASD severity [5]. Moreover, accurate evaluation of such skills is crucial to understanding the phenotypic heterogeneity as well as to building treatment strategies for optimal outcomes.

Common and rare genetic variants are major risk factors for ASD [6]. A meta-analysis summarizing several decades of twin studies estimated that the heritability of ASD ranges from 0.64–0.91 as demonstrated by the discrepancy in concordance rates for monozygotic and dizygotic twins with ASD of unknown cause [7]. The vast inherited component of ASD is supported by familial clustering of cases [8] and higher concordance rates in individuals with siblings who have ASD (2–8%) in comparison to the general population [9]. As such, decades of gene discovery efforts using genotyping microarray and next-generation sequencing uncovered common, rare, and *de novo* genetic variants which occur with higher frequency in individuals with ASD compared to the neurotypical population [10]. Several rare inherited and *de novo* copy number variants (CNVs) have previously been associated with ASD [11, 12]. Nonetheless, individuals with shared genetic risk factors do not present similar phenotypic profiles in the three core symptom domains of ASD [13].

The Research Domain Criteria (RDoC) was established by the National Institute of Mental Health (NIMH) to create a framework for research on pathophysiology that would ultimately inform classification schemes, with a focus on genetics, genomics, and neuroscience [14]. The idea was to introduce a categorization system parallel to the DSM-5, which links validated dimensions of functioning relevant to mental health to underlying biological systems. To narrow the gap between mental disorders and their genetic underpinnings, researchers consider endophenotypes. The commonly proposed models of endophenotypes as reviewed by Kendler and Neale are the liability-index (or “risk-indicator”) model and the mediational model [15]. The former mechanism postulates that risk for dichotomous mental disorders and continuous endophenotypes are correlated with a common set of genes. On the other hand, the latter model illustrates a causal pathway in which genetic variants influence endophenotypes, leading to a corresponding mental disorder. Although Kendler and Neale noted the stronger and more falsifiable nature of the mediational model, endophenotypes are explained most accurately with a bivariate or multivariate paradigm. Several endophenotypes of a disorder such as cognitive abnormalities and antisocial behavior in schizophrenia are a result of distinct components of genetic risk [16] while IQ and the other neurocognitive-related abilities are likely polygenic at the population level [17, 18].

Here we performed an endophenotype-wide association study to find genetic correlates for endophenotypes assessed by diverse instruments for ASD symptomology and associated cognitive deficits. We collected information regarding endophenotypes through severity scores of the core symptom domains of ASD and measures of neurocognitive development as evaluated by standard instruments and tests—ADI-R, ADOS, Repetitive Behavior Scale, Revised (RBS) [19], Social Responsiveness Scale version 2 (SRS) [20], Peabody Picture Vocabulary Test III (PPVT) [21], Raven’s Progressive Colored Matrices (RPCM) [22], Stanford-Binet Intelligence Scale, 5th edition (SB-5) [23], Vineland Adaptive Behavior Scale (VABS) [24], and head circumference (HC). These assessments are essential to evaluate positive and negative valences of ASD, as well as the related cognitive systems and social processes in the context of RDoC framework. With detailed endophenotypes and common variants (variant allele frequency (VAF) ≥ 5%)) extracted from whole-genome sequencing (WGS), we employed a linear mixed model (LMM) to sort out genetic substrates of phenotypic heterogeneity.

## Materials and methods

### Subjects

Family-based data were collected from all individuals who participated in the Autism Genetic Resource Exchange (AGRE), which compiles the WGS and phenotype data of families containing at least one individual diagnosed with ASD by the ADI-R and ADOS [25]. Although both instruments assess the three domains of ASD, they differ in format; the ADI-R is a structured caregiver interview and is shorter [26], while the ADOS involves observation of the examinee through a series of standardized scenarios [27]. The ADI-R was utilized to characterize individuals in our sample as Autism, Not Quite Autism (NQA), Broad Spectrum, or Not Met. Following previous methods [28], we classified individuals as “case” if they fell under the Autism or NQA categories while “unaffected” individuals were those who were characterized as Broad Spectrum or Not Met by the AGRE. In addition to ASD-specific diagnostic tests, participants were given an opportunity to complete additional phenotype evaluations. In the present study, the resulting scores were utilized in quantitative trait locus (QTL) analyses.

Our AGRE dataset consisted of 11,961 individuals with demographic and phenotypic information, including 3833 individuals with WGS data available. WGS data were collected through MSSNG and the Hartwell Autism Research and Technology Initiative (iHART) consortiums. MSSNG, a joint effort of Autism Speaks, University of Toronto, SickKids Hospital, and Google, is the largest collection of readily available WGS data for ASD researchers [29]. In its first phase of collection, MSSNG aimed to incorporate the phenotype scores and WGS data from individuals who were primarily part of the AGRE. iHART is distinct in that its collection of WGS data from AGRE individuals focuses on multiplex families [30]. Both repositories have allowed for the successful identification of novel candidate genes for ASD. A summary of the demographic data for the entire AGRE dataset as well as for individuals with WGS data that were subjected to the current study can be accessed in the Supplementary Table 1.

### Endophenotype scores

We analyzed 29 scores from eight instruments compiled in the AGRE dataset (ADI-R, ADOS, RBS, SRS, PPVT, RPCM, SB-5, and VABS) and HC. Each instrument covers one or more core symptom domains of ASD or neurocognitive development by age. ADI-R, ADOS, and SRS have components to estimate difficulties in social interaction. RRBs are scored in the ADI-R, ADOS, and RBS while deficits in verbal and nonverbal communication are mostly measured by the ADI-R and ADOS. General neurocognitive development is estimated by RPCM, PPVT, SB-5, and VABS. We summarize the instruments and endophenotype scores used in our study in the Supplementary Material. The number of individuals with scores for each phenotype measure (either in the entire AGRE dataset or with WGS data available) varied because of the differences in compliance and completion rates across phenotypic instruments (Supplementary Table 2). Among the anthropometric measurements, our analysis incorporated HC, which is a well-studied feature in the context of ASD and associated genetic conditions [31].

The ADI-R is a standardized, semi-structured interview administered by an experienced rater to caregivers of individuals suspected of having ASD. Effective for differentiating ASD from similar developmental disorders, the ADI-R is concerned with the participant’s development, social functioning, language acquisition, and RRBs. In our study, we used the 4 corresponding domain scores– Social, Verbal Communication, Nonverbal Communication, and Behavior. The ADOS is a standardized diagnostic test for ASD commonly used as a screening tool by school systems and clinicians. AGRE participants were administered ADOS Module 1, 2, or 3 at the discretion of a clinical psychologist according to their expressive language level. Through standardized scenarios, the test measures impairments in the domains of Social, Communication, Social-Communication, Stereotyped Behaviors and Restricted Interests (SBRIs; also referred to as RRBs) and Play (Module 1 only). We used all of the domain total scores available from each module (5 for Module 1, 4 for Modules 2 and 3) and the total scores for each module, resulting in a total of 16 phenotype scores. The RBS is a caregiver-informant questionnaire that quantifies various forms of RRBs that are characteristic of ASD [19]. Participants are evaluated on six subscales: stereotyped, self-injurious, compulsive, ritualistic, sameness, and restricted behaviors. The RBS Total Subscale score combines the subscale scores to provide a measure of RRB severity and was used for our analysis. The SRS is a widely accepted measure of social impairment in the realms of social awareness, social cognition, social communication, social motivation, and mannerisms [20]. We incorporated SRS total T-Scores in the current analysis.

The summary scores from four instruments—SB-5, PPVT, RCPM, and VABS—were used as indicators of age-adjusted neurocognitive development. For SB-5, Verbal IQ (VIQ), Nonverbal IQ (NVIQ), and Full-scale IQ (FSIQ) scores were used [23]. All three of these scores are age-normed (mean 100, standard deviation (SD) 15). To provide additional information about each participant’s neurocognitive development and encompass receptive vocabulary, we incorporated the PPVT Standard Score (mean 100, SD 15). The PPVT is an individually administered assessment of receptive lexical knowledge [21]. Of the three different versions recorded for the AGRE cohort, we chose ‘Version 3’ since it was used for most individuals with a reported PPVT score (1681 out of 2239). Consisting of a series of tasks in which participants are required to identify missing elements of matrix patterns, the RCPM is a measurement of nonverbal processing, fluid intelligence, and spatial reasoning [32]. We utilized Raw Total scores from the RCPM in our analyses [33]. The VABS is a semi-structured caregiver interview examining a participant’s adaptive behavior and living skills [24]. An individual’s level of functioning within the domains of communication, daily living skills, socialization, and motor skills are evaluated and used to derive the composite standard score–an age-normalized score (mean 100, SD 15) used for the current investigation.

### Factor analysis of endophenotype measurements

Exploratory factor analysis with 29 endophenotype scores was performed to find correlation structure among the scores and to explore whether 29 scores could be reduced to a smaller number of latent variables for association analysis with genotype data. Each module of ADOS was measured for different subgroups depending on the level of verbal communication. Thus, we used corresponding scores from three modules to represent ADOS scores for factor analysis. Multidimensional scaling was performed to check (dis)similarity of endophenotype measurements in a low-dimensional Euclidian space. The number of factors was determined using parallel analysis [34]. For these factors, we used an oblique rotation function that was implemented in the promax function in R stats package to calculate loadings of each measurement to rotated principal axes. Factor analysis was performed for 93 individuals with complete neurocognitive measurements, ADOS, SRS, RBS, and one of ADI-R modules using psych R library package [35].

### Genome-wide association analysis

Multi-sample variant call files (VCFs) were downloaded from the MSSNG (version db6, *N* = 9621) and iHART (version v01, *N* = 2308) project sites. Mean read depths were 30x or higher for both projects: ~40x and ~36x for MSSNG and iHART, respectively. Both projects applied stringent quality assurance criteria such as read depth coverage, variant call quality, agreement of genotype calls between WGS and microarray, and checking for sample relatedness and potential duplicates for the released data [29, 30]. The iHART project used the Genome Reference Consortium Human Build 37 (GRCh37) to process WGS while MSSNG used GRCh38. We used Picard LiftoverVcf tool to map all genomic variants in MSSNG data to GRCh37, then merged iHART and MSSNG VCF files on GRCh37 coordinates. We selected genotype data for the individuals with phenotype information (*N* = 3833). To create the variant call set for association analysis with endophenotype data, we applied the following inclusion criteria: (1) Hardy-Weinberg equilibrium at the threshold of *p* < 1 × 10^−6^, (2) bi-allelic variants of 0% genotype missing rate, and (3) VAFs between 5% and 95%. Further, we set linkage disequilibrium (LD) threshold *r*^*2*^ < 0.4 to remove correlated SNVs resulting 549,294 SNVs and 33,287 indels in autosomal chromosomes.

We applied a linear mixed model (LMM) to compute statistics for the association between genotype and each of endophenotype scores using BOLT-LMM v2.3.6 with --lmmForceNonInf option [36]. BOLT-LMM is an efficient implementation of the mixed-model association method via Bayesian modeling using a mixture-of-normals prior on effect sizes of genetic markers. We included age, sex, and top 10 principal components (PCs) as covariates. Most individuals had different subsets of endophenotype scores. Thus, top 10 PCs were recalculated to include the individuals with endophenotype score for each statistical model. To increase power to detect true positives, we used a genome-wide significance threshold of *p* < 5 × 10^−7^ to discover significant genetic loci [37, 38]. Further, we applied a threshold of *p* < 5 × 10^−8^ for a follow-up BOLT-LMM analysis including only European descents.

## Results

### Endophenotype scores

The diagnostic and neurocognitive measurements and the number of available participants for each measurement are listed in the Supplementary Table 2. Notably, the number of available endophenotype scores varied across individuals. We used all participants for each endophenotype score instead of selecting a subgroup (*N* = 509) with all endophenotype scores. Thus, each association test included a different number of individuals. For instance, the ADI-R Social domain score was available for 3746 individuals (includes 3386 probands and 358 unaffected siblings) while the SB-5 FSIQ score was available for 833 individuals (includes 681 probands and 146 unaffected siblings). We found significant differences (at a threshold of *p* < 0.01, Welch’s t-test) between endophenotype scores of our cohort compared to published scores for individuals with ASD: ADOS Module 3 SBRIs scores, ADI-R Verbal Communication Total score, SRS Total score, and RBS Total score (Supplementary Table 3). All the differences in endophenotype scores between our cohort and literature values reflect a higher severity of our samples, which is consistent with the inclusion of the large percentage of multiplex families within the AGRE for whom familial genetic liability could be higher [39].

**Fig. 1:**
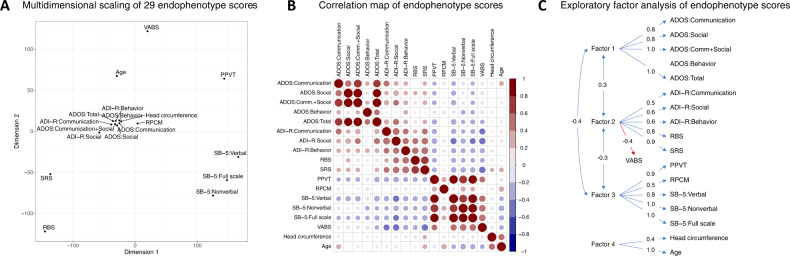
Correlation structure among 29 endophenotype scores and exploratory factor analysis. **A** Two-dimensional representation of 29 endophenotype measurements with multidimensional scaling using 93 individuals with all scores. **B** A pairwise correlation map between endophenotype scores and demographic variables. The color and size of each circle represent the Spearman correlation coefficient between variable pairs. **C** The four factors derived from exploratory factor analysis of endophenotype scores, age, and head circumference. The numbers on the arrow represent the correlation between latent variables or between latent variable and endophenotype scores.

For a subgroup with all endophenotype scores with WGS (*N* = 93), we examined the correlation structure of 29 endophenotype scores and performed exploratory factor analysis to check whether 29 scores could be reduced to a smaller number of latent variables. We used non-metric multidimensional scaling (MDS) with standardized scores to visualize the correlation structure of scores and found that the scores for measuring neurocognitive function were distinct from a cluster of ADOS and ADI-R domains scores and each of neurocognitive domains measured (Fig. 1A). For SB-5, verbal, non-verbal, and full-scale scores were in close proximity on Dimension 1 while the other scores were equally distant from SB-5 scores except for PPVT. Of note, RPCM was closer to ADOS and ADI-R domain scores compared to the other neurocognitive measurements. Pairwise-correlations between ADOS and ADI-R scores, RBS, and SRS were higher than those between neurocognitive measurements or between age and HC (Fig. 1B). Exploratory factor analysis discovered four latent factors. Factor 1 was associated with ADOS scores except for ADOS Behavioral total score (Fig. 1C). Factor 2 was associated with ADI-R measurements, RBS, and SRS. Of note, VABS that measures adaptive behavior was negatively correlated with factor 2. Overall, neurocognitive development measurements (i.e., SB-5, PPVT, and RPCM) were correlated with factor 3. Age was correlated with HC, and factor 4 represented age and HC. For all endophenotype scores, latent variables represented overall ASD severity (factors 1 and 2), neurocognitive development (factor 3) and demographic variables (factor 4). For association analysis with genotype, we included all individuals with a measurement instead of restricting to the subgroup of individuals with complete endophenotype scores to maximize power to detect true positive associations.

### Genetic substrates for core symptoms of autism spectrum disorder

**Fig. 2:**
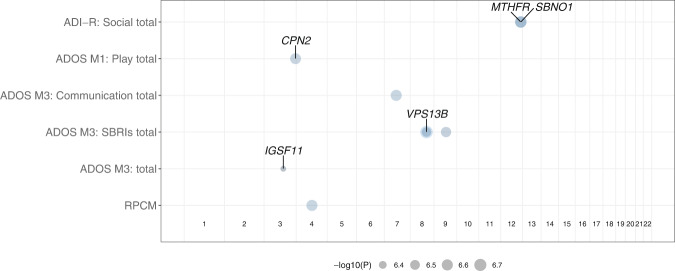
Overview of genomic loci associated with endophenotypic scores. Genome-wide association analysis with each of endophenotype scores highlights significant loci and genes. Horizontal axis indicates genomic position from chromosome 1 to chromosome 22 and each row in vertical axis is organized by test instruments and phenotypic scores. The phenotype scores with significantly associated loci are (top to bottom): Social score from ADI-R, Play score from ADOS Module 1, 3 scores from ADOS Module 3 (Communication, Stereotyped Behaviors and Restricted Interests, and Total scores) and RPCM Total score. Circles indicate genomic loci with *p* < 5 × 10^−7^, where the bigger the size the smaller the nominal *p*-value as indicated in the legend below x-axis. The genes that overlap with or in 250 kbps flanking region of each significant genomic loci are displayed next to the corresponding circles and significant loci in intergenic regions are displayed without an associated gene symbol.

We performed a genome-wide association test for each endophenotype score (*N* = 29) using BOLT-LMM. A total of 9 variants were significant at a genome-wide threshold of *p* < 5 × 10^−7^ (Table 1, Supplementary Figs. 1–6). We did not find an aggregation of loci for different endophenotype scores; instead, significant loci were scattered in autosomes (Fig. 2). ADI-R scores were associated with two significant loci: an intronic variant in the *MTHFR* gene and an upstream variant of the *SBNO1* gene—in chr12q24.31 for the Social domain. No significant association was found for other ADI-R domain scores such as Behavior or Communication (verbal or nonverbal). The association between the intron variant in *MTHFR* gene and ADI-R Social score was replicated when only European individuals were used in the analysis (*p* = 2.4 × 10^−7^).

**Table 1:** Common genetic variants associated with severity of core symptoms of ASD and neurocognitive measurements.

Phenotype Measures^1^	Name	Locus^2^	EA/OA^3^	Genes	All individuals				European individuals			
					Beta (s.e.)	*p*-value	FDR	*N*	Beta (s.e.)	*p*-value	FDR	*N*
ADI-R	Social	12:123737933	T/G	*MTHFR* (intron)	1.93 (0.374)	2.30 × 10^−7^	0.061	1881	2.11 (0.408)	2.40 × 10^−7^	0.13	1239
		12:123852510	A/G	*SBNO1* (upstream)	1.89 (0.366)	2.40 × 10^−7^	0.061		NS			
ADOS (Module 1)	Play	3:194058802	CATTTT/C	*CPN2* (downstream)	−0.82 (0.160	2.60 × 10^−7^	0.11	522	NS			311
ADOS (Module 3)	Communication	7:73420195	T/C	*ELN* (upstream)	0.91 (0.176)	2.50 × 10^−7^	0.12	600	0.96 (0.190)	4.50 × 10^−7^	0.25	440
	Stereotyped Behaviors and Restricted Interests	8:100650223	A/G	*VPS13B* (intron)	0.52 (0.102)	3.30 × 10^−7^	0.070	600	0.66 (0.121)	4.50 × 10^−8^	0.017	440
		8:100717925	G/T		0.57 (0.109)	1.80 × 10^−7^	0.070		0.71 (0.130)	4.50 × 10^−8^	0.017	
		9:74893024	A/G		−0.64 (0.126)	2.90 × 10^−7^	0.070		NS			
	Total score	3:118637716	A/C	*IGSF11* (intron)	2.76 (0.546)	4.30 × 10^−7^	0.20	598	NS			438
RPCM	Total score	4:97178781	A/G		1.8 (0.350)	2.50 × 10^−7^	0.24	1051	NS			697

^1^*ADI-R* Autism Diagnostic Interview-Revised, *ADOS* Autism Diagnostic Observation Schedule, *RBS* Repetitive Behavior Scale-Revised, *RPCM* Raven’s Progressive Colored Matrices.

^2^All genomic positions are given in Genome Reference Consortium Human Build 37 (GRCh37).

^3^*EA* Effect allele, *OA* Other allele, *s.e*.: standard error, *FDR* false discovery rate estimated using Benjamini-Hochberg procedure, NS: not significant at *p* < 5 × 10^−7^.

For the ADOS, six loci were associated with domain scores from Modules 1 and 3. ADOS Module 1 Play score was associated with a variant in 1.7 kb downstream from *CPN2* (*p* = 2.6 × 10^−7^). ADOS Module 3 Total score was associated with an intronic variant in *IGSF11* (*p* = 4.3 × 10^−7^). The immunoglobulin superfamily member 11 (IgSF11) is a dual-binding partner of the postsynaptic scaffolding protein PSD-95 and AMPA glutamate receptors and regulates excitatory synaptic plasticity [40]. A cross-disorder genome-wide association study compared 46,008 individuals with psychiatric disorders—attention-deficit/hyperactivity disorder, affective disorder, anorexia, ASD, bipolar disorder, or schizophrenia—to population controls, yielding the discovery that the *IGSF11* locus could be associated with multiple psychiatric disorders [41]. Moreover, a large-scale genome-wide association study (GWAS) of 1.1 million individuals for educational attainment discovered *IGSF11* as one of the genes enriched with significant SNVs [17].

ADOS Module 3 Communication total score was associated with 22 kb upstream variants of the *ELN* gene. CNVs in chromosome 7q11.23 encompassing the *ELN* gene are associated with syndromic neurodevelopmental disorders. 7q11.23 deletion is associated with Williams–Beuren syndrome (WBS; MIM#194050) [42] and 7q11.23 duplication was reported in ASD [43]. Patients with WBS show strengths in language, music, facial processing [42], and sociability while severe neurocognitive impairment in visuospatial construction is observed [44]. It is not known whether the *ELN* gene is involved in the social and neurocognitive phenotype of WBS and some ASD cases [45]; however, the *ELN* gene could be a candidate for further investigation. The same association was replicated in the association analysis with European descents.

**Fig. 3:**
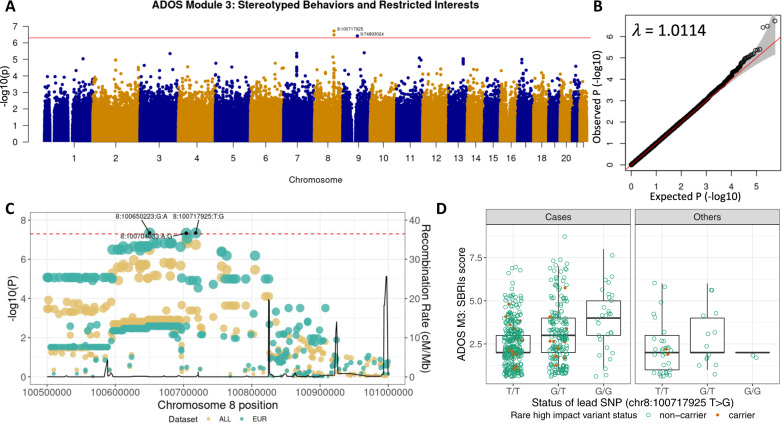
Association between ADOS Module 3 stereotyped behaviors and restricted interests total score and variants in the *VPS13B* gene. **A** The Manhattan plot of the quantitative locus analysis (QTL) results. The top-significant variants with *p* < 5 × 10^−7^ in each chromosome are labeled by their genomic loci as chromosome:base-position. The horizontal line represents the threshold of *p* = 5 × 10^−7^. The *y*-axis represents *p*-value in -log_10_ scale for genome-wide association test with SBRIs score based on linear mixed model using BOLT-LMM software. **B** A quantile-quantile plot and the genomic inflation factor (λgc) estimated for genome-wide association analysis (GWAS) with ADOS Module 3 Stereotyped Behaviors and Restricted Interests score. No significant inflation is found for the association test statistics (λgc = 1.0114). **C** Regional plot showing a significant quantitative trait locus around the *VPS13B* gene (located in chromosome 8:100025494–100889814). The horizontal dotted line represents the genome-wide significance level (*p* = 5 × 10^−8^). Each dot represents variant tested for association with its size proportional with its -log_10_(*p*-value). The yellow dots show results using all available individuals (*N* = 600), while the green dots with European individuals only (*N* = 440). The line graph shows the recombination rates (cM/Mb). **D** Distribution of SBRIs scores by the status of lead variant (8:100717925 T > G, or rs2510202) in the *VPS13B* gene. Individuals carrying variant allele (G-allele) show higher SBRIs score in ADOS Module 3. Also, individuals with rare and high impact variants in the *VPS13B* gene (brown filled dots) do not present extreme SBRIs scores than non-carriers (green circle).

The strongest association was found for the *VPS13B* gene. SBRIs total score in ADOS Module 3 was associated with three loci across autosomal chromosomes with two loci in *VPS13B* (Fig. 3A). We calculated the genomic inflation factor (λgc) to check the potential confounding effects from population structure and hidden variables. For the test statistics of ADOS Module 3 SBRIs, λgc was 1.0114, which did not suggest a departure from the theoretical chi-square statistics (Fig. 3B). We also performed the same analysis including only European descents and found that two variants in the *VPS13B* gene were significant for the same score at a genome-wide threshold of *p* < 5 × 10^−8^ (Fig. 3C). Next, we checked whether the individuals with rare and high impact variants in *VPS13B* would have extreme endophenotype scores. SBRIs Total score in ADOS Module 3 was correlated with rs2510202: G-allele (NC_000008.10:g.100717925T>G) while the individuals with rare and high impact variants in this gene did not have extreme scores (Fig. 3D). The *VPS13B* (vacuolar protein sorting-associated protein 13B) gene encodes a potential transmembrane protein that mediates vesicle transport through the Golgi apparatus and is involved in protein sorting within the cell. Mutations in *VPS13B* are causally associated with Cohen syndrome (MIM# 216550), which is an autosomal recessive disorder characterized by microcephaly, facial dysmorphism, hypotonia, intellectual disability, and intermittent neutropenia [46–48]. Individuals with biallelic null mutations in *VPS13B* present with a phenotypic profile characteristic of ASD, but across individuals, a wide array of symptom severity is observed with missense mutations in this gene [49]. Moreover, a homozygous frameshift mutation in *VPS13B* was found in an ASD case with mild dysmorphic features and microcephaly [50]. The molecular mechanism of Cohen syndrome is not known yet while the function of VPS13B in tethering of endosomes [51] and autophagy in neuronal cells are recently reported [40]. In addition to the intronic variants in *VPS13B*, an intergenic variant in chromosome 9 (27k downstream of the *GDA* (Guanine Deaminase) gene) was found for SBRIs score in ADOS Module 3.

### Genetic substrates for cognitive systems in autism

Multiple genetic loci were associated with domain scores of ADOS and ADI-R; however, we did not find strong associations between genotype and neurocognitive measurements except for the association between RPCM and an intergenic variant in 400 kb downstream from the *PDHA2* gene on chr4q22.3 (*p* = 2.5 × 10^−7^).

## Discussion

Gene discovery efforts with genotyping microarrays, whole-exome sequencing (WES), and WGS have been successful to catalog candidate genes for ASD [52]. GWASs with larger sample sizes have had limited success in identifying the role of common genetic variants in ASD [53] while large-scale sequencing projects are responsible for discoveries of ASD candidate genes [54]. As such, *de novo* variants with high impacts on gene function have been prioritized for causal genes and treatment targets [55]. Independent studies on multiple cohorts consistently found that genetic underpinnings of ASD would be polygenic and comprise both common and rare variants in hundreds of candidate genes [56]. Yet genetic discovery has not been translated into molecular pathways and brain circuits that may explain phenotypic heterogeneity in core symptoms, neurocognitive development, and comorbidities in individuals with ASD [2]. Therefore, uncovering genetic substrates for behavioral and cognitive endophenotypes will further define molecular diagnosis and prioritize treatment targets for ASD. Recently, Warrier and colleagues reported the genetic correlates of heterogeneous phenotype of ASD [57]. Factor analysis was performed to extract six latent variables from RBS and Social Communication Questionnaire—Lifetime version (SCQ). Then, a linear model was used to calculate the variance of phenotype scores (e.g., six latent variables, core ASD symptoms, and neurocognitive measurements) explained by polygenic risk scores (PRSs) and the number of high impact *de novo* variants. Interestingly, types of variants had differential impacts on the core symptoms and neurocognitive development measurements. This study illustrated the complexity of underlying genetic correlates with core symptoms of ASD and neurocognitive development; however, candidate genes for each of core symptom domains were not reported. In fact, to achieve an adequate power to detect genetic effects of high impact rare and *de novo* variants on endophenotype, tens of thousands of cases would be required [58]. Rare variant association studies for endophenotypes will be plausible as ongoing efforts such as the Autism Sequencing Consortium [59] and MSSNG continue to generate WES and WGS data. Nonetheless, harmonizing endophenotype data from multiple datasets will be challenging.

In the current study, we focused on common variants with VAFs greater than 5% to discover genetic loci associated with the severity of core symptoms and neurocognitive development. Interestingly, the common genetic variants in the *VPS13B* gene—a disease-causing gene for Cohen syndrome—were associated with a core symptom of ASD. Moreover, the association between the common variants in *VPS13B* and ADOS Module 3 SBRIs Total score achieved a genome-wide significance of 5 × 10^−8^ when participants of European descent were tested. Allelic heterogeneity is associated with phenotypic variability in rare and common diseases. As such, an extreme phenotypic spectrum is often observed for Mendelian disorders with rare and high impact genetic variants such as loss-of-function and nonsense variants while common variants in the same gene affect a larger group of individuals including subclinical phenotypes. For instance, familial hypercholesterolemia (FH, MIM# 143890) is a genetic condition for which rare and common genetic risk factors are associated with a spectrum of phenotypic severity (i.e., elevated low-density lipoprotein cholesterol (LDL-C) levels) [60]. The *LDLR, APOB*, and *PCSK9* genes are associated with the phenotypic spectrum of FH. Rare homozygous null mutations in *LDLR* results in the highest LDL-C levels while heterozygous missense variants in *APOB* and gain-of-function variants in *PSCK9* are associated with a moderate increase in LDL-C levels. Of note, we did not find extreme phenotype scores for the individuals with rare and high impact variants in *VPS13B*.

Understanding the biological basis of phenotypic heterogeneity is essential to discover treatment biomarkers [2]. RRBs comprise one of the core symptom domains of ASD; however, these behaviors are observed in multiple neuropsychiatric conditions (e.g., schizophrenia, bipolar disorder, obsessive-compulsive disorder, drug addiction, L-DOPA-induced dyskinesia, and Huntington’s disease) [61]. Moreover, RRBs are observed in diverse genetic syndromes (e.g., Prada-Willie syndrome, Fragile X syndrome, and Rett syndromes) and are likely to be associated with multiple neurotransmitters such as GABA, dopamine, glutamate, and serotonin [62]. At a brain circuit level, the cortico-striatal pathway is associated with RRBs [63]. Behavioral approaches are used to treat RRBs, and several pharmacological treatments have been effective in reducing these behaviors in ASD. Therefore, RRBs are treatment targets; however, biological pathways associated with RRBs remain undiscovered. Thus, our discovery of *VPS13B* as a putative genetic correlate for RRBs is intriguing.

Despite our compilation of one of the largest genotype-endophenotype datasets for ASD, the sample size was moderate for several endophenotypes within our study. The ADI-R Nonverbal Communication Total was associated with a noteworthy locus—intronic regions of *SEMA3E*— at chromosome 7q21.11 for which structural variations such as microdeletions have been reported in ASD as well as other disorders implicating developmental delays. No SNV was significant at *p* < 5 × 10^−7^ while 18 SNVs were found at *p* < 5 × 10^−6^ (Supplementary Fig. 7). While previous findings related to the *SEMA3E* gene have not been specific to ASD, chromosome 7q21.11 microdeletions involving this gene are described in patients with CHARGE syndrome (MIM# 214800) for whom the behavioral phenotype of Autism is frequently reported [64]. *SEMA3E* is a semaphorin, which is a class of proteins that interact as ligands with plexin receptors to regulate axon growth. Specifically, *SEMA3E* acts as both repellent and attractant depending on the presence of Neurophiln-1 [56]. A larger sample size will be required to validate the association between ADI-R Nonverbal Communication Total score and genetic variants in *SEMA3E* gene at a genome-wide threshold. Likewise, we discovered that two loci were associated with HC at *p* < 5 × 10^−6^ (Supplementary Fig. 8). These loci were mapped to the intronic region of *NKAIN3* gene, which encodes the Sodium/Potassium Transporting ATPase Interacting 3 protein. *NKAIN3* encompasses a risk allele for dyslexia [65] and is a known candidate gene for Dravet syndrome (MIM# 607208), which is a disorder characterized by an infantile-onset epileptic encephalopathy, intellectual disability, and refractory seizures [66].

The current study had a few limitations. Firstly, the loci discovered for endophenotype scores require a replication in another cohort. The AGRE participants are primarily multiplex families, incorporating individuals with pervasive developmental disorders (PDD) and Asperger syndrome as diagnosed by experts using the ADI-R and ADOS. Multiplex families with ASD can display higher genetic burdens compared to sporadic cases; however, our analysis aimed to find genetic substrates of phenotype tests covering core symptom domains and neurocognitive development rather than to discover associations between ASD and neurotypical controls. A similar study can be performed for different cohorts to validate the associations from the current study. Secondly, sample size for each of available endophenotype scores was moderate, leading to the discovery of loci with small effect sizes. For instance, ADOS Module 2 scores were available for a subgroup of our cohort (*N* = 311) while 1881 individual scores were available for the social and behavior domains of ADI-R. Thirdly, our analysis strategy was susceptible to the potential increase in type I error. We did not use PRS to correlate with endophenotype scores since we aimed to discover candidate genes for core symptoms of ASD. To reduce spurious correlations, a subset of markers with VAF ≥ 5% in linkage disequilibrium were selected from WGS data. Subsequently, we performed GWASs using a subgroup of European ancestry and replicated the original findings with *p* < 5 × 10^−8^ for the markers in *VPS13B*. Fourthly, genotype-phenotype associations found in our study may be valid for individuals with ASD and their family members. As unaffected siblings were included in the analysis, some associations with ADI-R and ADOS scores might indicate the genotype difference between affected and unaffected individuals.

In summary, we used common genetic variants and endophenotype scores to successfully perform a QTL analysis that extends previous candidate gene discovery for ASD by unveiling the genetic basis of core symptoms and neurocognitive deficits. Although specific genes, molecular mechanisms, and brain circuits implicated in the disorder remain undiscovered, understanding some of the biological substrates that underlie specific symptoms is valuable to define target symptoms for treatments and, thus, to develop therapeutic approaches. To this end, we aimed to discover the genetic basis of the core symptom domains and neurocognitive development in ASD using rich phenotype information and WGS data from the AGRE. Notably, a causal gene for syndromic ASD—*VPS13B* and Cohen syndrome—that was previously discovered by a family study was associated with the severity of a core ASD symptom. It is possible, therefore, that *VPS13B* could be responsible for a specific trait (i.e., RRBs), which constitutes the symptomatology of ASD rather than the disorder itself. Further studies are required to replicate our findings and to understand the genetic impacts on molecular pathways, brain circuits, and the phenotype spectrum in the context of RDoC framework.

## Supplementary information

Supplementary Material
