# Supplementary material for: Quantitative trait locus analysis for endophenotypes reveals genetic substrates of core symptom domains and neurocognitive function in autism spectrum disorder
Source: Transl Psychiatry. 2022 Sep 24;12:407. doi: 10.1038/s41398-022-02179-3 (PMC9509384; doi:10.1038/s41398-022-02179-3)

Supplementary Information for

Quantitative trait loci analysis for endophenotypes reveals genetic substrates of core symptom domains and neurocognitive function in autism spectrum disorder

Authors:
In-Hee Lee, Ph.D.,[1] Ekaterina Koelliker,[2] Sek Won Kong, M.D.[1,3]

Affiliations:
1. Computational Health Informatics Program, Boston Children's Hospital, Boston, MA 02115, USA
2. Psychology Department, Colby College, Waterville, ME 04901, USA
3. Department of Pediatrics, Harvard Medical School, Boston, MA 02115, USA

Corresponding Author:
Sek Won Kong, M.D.
sekwon.kong@childrens.harvard.edu
40 Park Drive, LM5528.4
Boston, MA 02215, USA
Phone: 1-617-919-2689

List of Supplementary Information

Supplementary Material. Descriptions of Phenotype Measures

Supplementary Tables.

Supplementary Table 1. Summary of demographic information for AGRE dataset.

Supplementary Table 2. Phenotype measures used for association study and data availability.

Supplementary Table 3. Comparison between phenotype scores of our cohort and published scores for individuals with ASD.

Supplementary Figures.

Supplementary Figures 1-6. Manhattan and quantile-quantile plots from GWAS results based on linear mixed model for each phenotype score in **Table 1**.

Supplementary Figure 7. Regional plot around the intronic region on *SEMA3E* for ADI-R Communication - Nonverbal Total score.

Supplementary Figure 8. Regional plot around the intronic region on *NKAIN3* for maximum head circumference.

Supplementary Material. Descriptions of Phenotype Measures

Autism Diagnostic Observation Schedule, Generic (ADOS-G; Lord et al. 2000)

ADOS is a standardized diagnostic test for ASD commonly used as a screening tool by school systems and clinicians. The assessment involves direct observations of the participant under controlled conditions in the absence of caregivers. Participants typically complete one of four available modules that is most suitable for their age and functional level. In our population, participants were administered Module 1, 2, or 3 at the discretion of a clinical psychologist. Through standardized scenarios, participants encounter minor obstacles, and their reactions serve as scorable behaviors to measure social and communication deficits. Specifically, the test measures impairments in the domains of social, communication, social-communication, play (Module 1 only) and restrictive and repetitive behaviors. Participants receive an ADOS classification of ASD if they meet cut-off scores on all the domains.[1] Calibrated severity scores (CSS) that incorporate the four domains were used in the present analyses because they can be compared across modules.[2] For modules 2 and 3, empirically determined threshold scores are widely used by psychologists to classify autism status for individuals.[3]

Autism Diagnostic Interview, Revised (ADI-R; Lord, Rutter, & Le Couteur, 2003)

The ADI-R is a standardized, semi-structured interview administered by an experienced rater to a caregiver of participants suspected of having ASD. Effective for differentiating ASD from similar developmental disorders, the ADI-R is concerned with the participant's development, social functioning, language acquisition, and restricted behaviors. The four ADI-R calculated total scores (social, nonverbal communication, verbal communication, and behavior) that comprise the deficits that are characteristics of autism were used in our analyses.[4] For the social, communication, and behavior domains, individuals with scores above well-established cut-off scores (10, 8, and 3, respectively) are considered to have Autistic Disorder.[5]

Social Responsiveness Scale, Second Edition (SRS-2; Constantino & Gruber, 2012)

The SRS-2 is a 65-item questionnaire completed by a parent or teacher that is most widely understood to assess social impairment (e.g. social awareness, social cognition, social communication, social motivation, and mannerisms).[6] The gender-adjusted T-score total generated by the SRS serves as the most reliable assessment of social deficits for ASD.[7] In support of the use of the T-score total score rather than the subscale scores, the creators of the evaluation emphasize that the subscales should be interpreted cautiously. For this reason, our analyses only incorporate the T-score total. Scores on this domain that are greater than 76 are considered to meet threshold for severe social impairment.[6]

Repetitive Behavior Scale, Revised (RBS-R; Bodfish et al. 2000)

The RBS-R is an empirically derived, quantitative measure of various forms of restricted repetitive behavior (RRB) that is characteristic of ASD. The caregiver-informant questionnaire allows researchers to evaluate participants on six subscales: stereotyped behavior, self-injurious behavior, compulsive behavior, ritualistic behavior, sameness behavior, restricted behavior, and an overall score.[8] Previous literature has utilized the RBS total score to measure severity of the described behaviors in ASD.[9]

Raven's Progressive Colored Matrices (RPCM; Raven et al. 1995)

Consisting of a series of tasks in which participants are required to identify missing elements of matrix patterns, the Ravens serves as a paramount measurement of nonverbal processing, fluid intelligence, and spatial reasoning.[10] The assessment is individually administered, norm-referenced, and accepted as a measurement of nonverbal IQ.[11] Our study utilized raw total scores in the analyses because, as reported in the literature, standard scores are not available from the manual.[12]

Peabody Picture Vocabulary Test III (PPVT; Dunn & Dunn, 1997)

The PPVT is an individually administered assessment of receptive lexical knowledge. During the test, individuals must select one of four pictures that corresponds to a word that is verbalized by the examiner.[13]d Although the test also yields a mental age and percentile rank, our study utilizes the PPVT standard score (*M*=100, *SD*=15) to encompass receptive vocabulary, which is consistent with previous genotype-phenotype studies.[14] Moreover, correlational analyses have demonstrated that this score can serve as a proxy for verbal IQ.[15] Values below 70 for the standard PPVT score indicate impairment in language development.[16]

Stanford-Binet Intelligence Scales: Fifth Edition (SB-5; Roid, 2005)

The individually administered SB-5 quantifies the cognitive abilities and intelligence of clinical and nonclinical populations. Subscores in both the verbal and nonverbal domains are calculated for fluid reasoning, knowledge, quantitative reasoning, visual/spatial reasoning, and working memory, which allows for the separation of verbal and nonverbal total scores.[17] The total scores from these two realms can be combined to yield the full scale IQ (FSIQ), which is used in addition to verbal IQ (VIQ) and nonverbal IQ (NVIQ) in the present study. All three of these scores are age-normed (*M*=100, *SD*=15). Values below 70 for full scale, nonverbal, and verbal IQ indicate intellectual disability.[18]

Vineland Adaptive Behavior Scales (VABS; Sparrow, Balla, & Cicchetti, 1984)

The VABS is a semi-structured caregiver interview examining a participant's adaptive behavior and living skills. Functioning within the domains of communication, daily living skills, socialization and motor skills are evaluated and used to derive the Adaptive Behavior Composite (ABC) score.[19] For the purposes of the current investigation, we used the ABC as it is an age-normalized score (*M*=100, *SD*=15). Participants scoring below 85 on the ABC are classified as having an intellectual disability.[20]

References

1. Lord, C. *et al.* The Autism Diagnostic Observation Schedule—Generic: A Standard Measure of Social and Communication Deficits Associated with the Spectrum of Autism. *J. Autism Dev. Disord.* **30**, 205–223 (2000).
2. Solomon, M. *et al.* Using the NIH Toolbox to Assess Cognition in Adolescents and Young Adults with Autism Spectrum Disorders. *Autism Res. Off. J. Int. Soc. Autism Res.* **14**, 500–511 (2021).
3. GOTHAM, K. *et al.* A Replication of the Autism Diagnostic Observation Schedule (ADOS) Revised Algorithms. *J. Am. Acad. Child Adolesc. Psychiatry* **47**, 642–651 (2008).
4. Lord, C., Rutter, M. & Le Couteur, A. Autism Diagnostic Interview-Revised: A revised version of a diagnostic interview for caregivers of individuals with possible pervasive developmental disorders. *J. Autism Dev. Disord.* **24**, 659–685 (1994).
5. Le Couteur, A., Haden, G., Hammal, D. & McConachie, H. Diagnosing Autism Spectrum Disorders in Pre-school Children Using Two Standardised Assessment Instruments: The ADI-R and the ADOS. *J. Autism Dev. Disord.* **38**, 362–372 (2008).
6. Constantino, J. N. & Gruber, C. P. *Social responsiveness scale: SRS-2*. (Western Psychological Services, 2012).
7. Bruni, T. P. Test Review: Social Responsiveness Scale–Second Edition (SRS-2). *J. Psychoeduc. Assess.* **32**, 365–369 (2014).
8. Bodfish, J. W., Symons, F. J. & Lewis, M. H. *The repetitive behavior scale*. (Western Carolina Center Research Reports, 1999).
9. Nayar, K. *et al.* Elevated Polygenic Burden for Autism Spectrum Disorder Is Associated With the Broad Autism Phenotype in Mothers of Individuals With Autism Spectrum Disorder. *Nov. Neurodev. Disturb. Autism Risk* **89**, 476–485 (2021).
10. Raven, J. C., Court, J. H. & Raven, J. *Manual for Raven's Progressive Matrices and Vocabulary Scales*. (Oxford Psychologists Press, 1995).
11. Mottron, L. Matching Strategies in Cognitive Research with Individuals with High-Functioning Autism: Current Practices, Instrument Biases, and Recommendations. *J. Autism Dev. Disord.* **34**, 19–27 (2004).
12. Fernández-Alcaraz, C. & Carvajal, F. Neuropsychological profile of adults with Down syndrome and moderate intellectual disability. *Res. Dev. Disabil.* **107**, 103781 (2020).
13. Dunn, L. M. & Dunn, L. M. *Peabody picture vocabulary test-III*. (American Guidance Service, 1997).
14. Goin-Kochel, R. P. *et al.* The MTHFR 677C→T polymorphism and behaviors in children with autism: exploratory genotype–phenotype correlations. *Autism Res.* **2**, 98–108 (2009).
15. Krasileva, K. E., Sanders, S. J. & Bal, V. H. Peabody Picture Vocabulary Test: Proxy for Verbal IQ in Genetic Studies of Autism Spectrum Disorder. *J. Autism Dev. Disord.* **47**, 1073–1085 (2017).
16. Stockman Ida J. The New Peabody Picture Vocabulary Test—III: *Lang. Speech Hear. Serv. Sch.* **31**, 340–353 (2000).
17. Roid, G. H. *Stanford Binet Intelligence Scales for Early Childhood*. (Pro-ed, 2005).
18. *Intellectual Disability: Definition, classification, and systems of support*. (American Association on Intellectual and Developmental Disability (AAIDD), 2010).
19. Sparrow, S. S., Balla, D. A., Cicchetti, D. V. & Harrison, P. L. *Vineland adaptive behavior scales*. (American Guidance Service, 1984).

20. Green, S. A., Berkovits, L. D. & Baker, B. L. Symptoms and development of anxiety in children with or without intellectual disability. *J. Clin. Child Adolesc. Psychol. Off. J. Soc. Clin. Child Adolesc. Psychol. Am. Psychol. Assoc. Div. 53* **44**, 137–144 (2015).

Supplementary Table 1. Summary of demographic information for AGRE dataset.

(A) All individuals with phenotype data (N=11,961).

Diagnosis	Female	Male	Unknown/Missing	Mean Age in Years (SD)
Proband-Autism	650	2,629	0	8.5 (4.78)
Proband-NQA	33	95	0	8.1 (5.38)
Sibling-BroadSpectrum	77	174	0	8.5 (5.53)
Sibling-Not Met	73	38	0	10.1 (3.48)
Sibling-Unaffected	802	705	0	10.4 (6.77)
Parents	2,048	2,093	0	40.2 (6.94)
Unknown/Missing/Affected Parents	642	695	1,207	N/A
**Total**	**4,325**	**6,429**	**1,207**	

(B) Individuals with both phenotype and WGS data (N=3,833).

Diagnosis	Female	Male	Unknown/Missing	Mean Age in Years (SD)
Proband-Autism	502	1,188	0	8.5 (4.43)
Proband-NQA	26	43	0	7.6 (3.95)
Sibling-BroadSpectrum	49	86	0	8.3 (5.52)
Sibling-Not Met	4	1	0	11.2 (5.17)
Sibling-Unaffected	128	103	0	9.7 (5.61)
Parents	837	793	0	39.9 (6.82)
Unknown/Missing/Affected Parents	18	18	37	N/A
**Total**	**1,564**	**2,232**	**37**	

Supplementary Table 2. Phenotype measures used for association study and data availability.

Phenotype Measures[1]			Domains of ASD assessed by phenotype tests				Individuals with phenotype data (N=11,961)[2]					
			Social	Behavioral	Communication	Development	Proband		Sibling		Other	Total
							Female	Male	Female	Male		
ADOS	Module 1	Communication Total			✓		210 (165)	803 (342)	6 (5)	17 (10)		1,036 (522)
		Social Total	✓				210 (165)	803 (342)	6 (5)	17 (10)		1,036 (522)
		Communication+Social Total	✓		✓		210 (165)	803 (342)	6 (5)	17 (10)		1,036 (522)
		Play Total	✓		✓		210 (165)	803 (342)	6 (5)	17 (10)		1,036 (522)
		Stereotyped Behaviors and Restricted Interests Total		✓			210 (165)	803 (342)	6 (5)	17 (10)		1,036 (522)
		Total Score	✓	✓	✓		207 (162)	799 (339)	6 (5)	17 (10)		1,029 (515)
	Module 2	Communication Total			✓		110 (87)	448 (207)	16 (4)	28 (13)		602 (311)
		Social Total	✓				110 (87)	448 (207)	16 (4)	28 (13)		602 (311)
		Communication+Social Total	✓		✓		110 (87)	448 (207)	16 (4)	28 (13)		602 (311)
		Stereotyped Behaviors and Restricted Interests Total		✓			110 (87)	448 (207)	16 (4)	28 (13)		602 (311)
		Total Score	✓	✓	✓		110 (87)	447 (206)	16 (4)	28 (13)		601 (310)
	Module 3	Communication Total			✓		199 (166)	796 (386)	77 (22)	93 (26)		1,165 (600)
		Social Total	✓				199 (166)	796 (386)	77 (22)	93 (26)		1,165 (600)
		Communication+Social Total	✓		✓		199 (166)	796 (386)	77 (22)	93 (26)		1,165 (600)
		Stereotyped Behaviors and Restricted Interests Total		✓			199 (166)	796 (386)	77 (22)	93 (26)		1,165 (600)
		Total Score	✓	✓	✓		199 (166)	795 (385)	77 (22)	92 (25)		1,163 (598)
ADI-R	Social Total		✓				675 (524)	2,711 (1,221)	149 (52)	209 (84)	2	3,746 (1,881)
	Communication - Verbal Total				✓		476 (373)	1,909 (910)	141 (47)	184 (68)	2	2,712 (1,398)
	Communication - Nonverbal Total				✓		199 (151)	802 (311)	8 (5)	25 (16)		1,034 (483)
	Repetitive Behaviors and Stereotyped Patterns of Behavior Total			✓			675 (524)	2,711 (1,221)	149 (52)	209 (84)	2	3,746 (1,881)
SRS	Total T-Score		✓				312 (242)	1,266 (524)	357 (77)	314 (66)	70 (7)	2,319 (916)
RBS	Total Subscale Score			✓			190 (154)	760 (364)	226 (43)	175 (35)	46 (5)	1,397 (601)
RPCM	Raw Total Score					✓	378 (306)	1,472 (663)	119 (37)	132 (43)	8 (2)	2,109 (1,051)
PPVT	Standard Score (version 3)				✓	✓	284 (219)	1,153 (404)	117 (32)	120 (29)	7 (1)	1,681 (685)
SB-5	Nonverbal IQ					✓	135 (112)	561 (286)	82 (14)	64 (17)	6 (4)	848 (433)
	Verbal IQ					✓	135 (112)	547 (283)	83 (14)	64 (17)	6 (4)	835 (430)
	Full-scale IQ					✓	134 (111)	547 (283)	82 (14)	64 (17)	6 (4)	833 (429)
VABS	Standard Score					✓	450 (359)	1,755 (751)	116 (36)	132 (36)	1	2,454 (1,182)
Head Circumference							418 (335)	1,552 (693)	429 (102)	369 (92)	1 752 (783)	4,520 (2,005)

[1] ADOS: Autism Diagnostic Observation Schedule, ADI-R: Autism Diagnostic Interview-Revised, SRS: Social Responsiveness Scale, RBS: Repetitive Behavior Scale-Revised, RPCM: Raven's Progressive Colored Matrices, PPVT: Peabody Picture Vocabulary Test, SB-5: Stanford-Binet Intelligence Scale, VABS: Vineland Adaptive Behavior Scale

[2] Counts among individuals with both phenotype and WGS (N=3,833) are shown in parentheses.

Supplementary Table 3. Comparison between phenotype scores of our cohort and published scores for individuals with ASD.

Phenotype Measures[1]			Comparison significance (p-value)[2]	Reference for baseline scores
ADOS	Module 1	Communication Total	0.482	Ref [1]
		Social Total	0.1428	
		Communication+Social Total	0.2069	
		Play Total	0.02648	
		Stereotyped Behaviors and Restricted Interests Total	0.823	
		Total Score	0.3308	Ref [2]
	Module 2	Communication Total	0.4479	Ref [1]
		Social Total	0.8043	
		Communication+Social Total	0.6358	
		Stereotyped Behaviors and Restricted Interests Total	0.8891	
		Total Score	0.6478	Ref [2]
	Module 3	Communication Total	0.6171	Ref [1]
		Social Total	0.013	
		Communication+Social Total	0.01828	
		Stereotyped Behaviors and Restricted Interests Total	$< 2.2 \times 10^{-16}$	
		Total Score	0.01857	Ref [2]
ADI-R	Social Total		0.06108	Ref [1]
	Communication – Verbal Total		0.005941	
	Communication – Nonverbal Total		0.3202	
	Repetitive Behaviors and Stereotyped Patterns of Behavior Total		0.1398	
SRS	Total T-Score		2.64×10^{-14}	Ref [3]
RBS	Total Subscale Score		2.07×10^{-08}	Ref [4]
RPCM	Raw Total Score		0.8326	Ref [5]
PPVT	Standard Score (version 3)		0.4997	Ref [6]
SB-5	Nonverbal IQ		0.08193	Ref [7]
	Verbal IQ		0.9245	
	Full-scale IQ		0.4304	
VABS	Standard Score		0.04886	Ref [8]

[1] ADOS: Autism Diagnostic Observation Schedule, ADI-R: Autism Diagnostic Interview-Revised, SRS: Social Responsiveness Scale, RBS: Repetitive Behavior Scale-Revised, RPCM: Raven's Progressive Colored Matrices, PPVT: Peabody Picture Vocabulary Test, SB-5: Stanford-Binet Intelligence Scale, VABS: Vineland Adaptive Behavior Scale

[2] P-value from Welch's t-test comparing mean from our cohort and published baseline scores.

Ref [1]. Gotham *et al.*, J Am Acad Child Adolesc Psychiatry, 2008 (PMID: 18434924).

Ref [2]. Janvier *et al.*, J Autism Dev Disord, 2022 (PMID: 33826039).

Ref [3]. Coon *et al.*, Mol Autism, 2010 (PMID: 20678250).

Ref [4]. Lam and Aman, J Autism Dev Disord, 2007 (PMID: 17048092).

Ref [5]. Goharpey, Crewther, and Crewther, Res Dev Disabil, 2013 (PMID: 24139715).

Ref [6]. Joseph, McGrath, and Tager-Flusberg, Dev Neuropsychol, 2005 (PMID: 15843102).

Ref [7]. Coolican, Bryson, and Zwaigenbaum, J Autism Dev Disord, 2008 (PMID: 17410416).

Ref [8]. Bishop et al, J Autism Dev Disord, 2013 (PMID: 23065116).

Supplementary Figure Legends.

Supplementary Figures 1-6. Manhattan and quantile-quantile plots from GWAS results based on linear mixed model for each phenotype score in **Table 1**. In Manhattan plots, the horizontal lines represent P-value threshold of 5×10^{-7}. The variants with top P-value above the threshold in each chromosome are labelled by their genomic loci. The quantile-quantile plots are shown next to the corresponding Manhattan plots. Top plots for GWAS results using all available individuals, and bottom plots with European individuals only.

Supplementary Figure 7. Regional plot around the intronic region on *SEMA3E* for ADI-R Communication - Nonverbal Total score.

Supplementary Figure 8. Regional plot around the intronic region on *NKAIN3* for maximum head circumference.

Supplementary Figure 1. Manhattan and quantile-quantile plots for ADI-R Social score. In Manhattan plots, the horizontal lines represent P-value threshold of 5×10^{-7}. The variants with top P-value above the threshold in each chromosome are labelled by their genomic loci. The quantile-quantile plots are shown next to the corresponding Manhattan plots. (top) GWAS results using all available individuals. (bottom) Results with European individuals only.

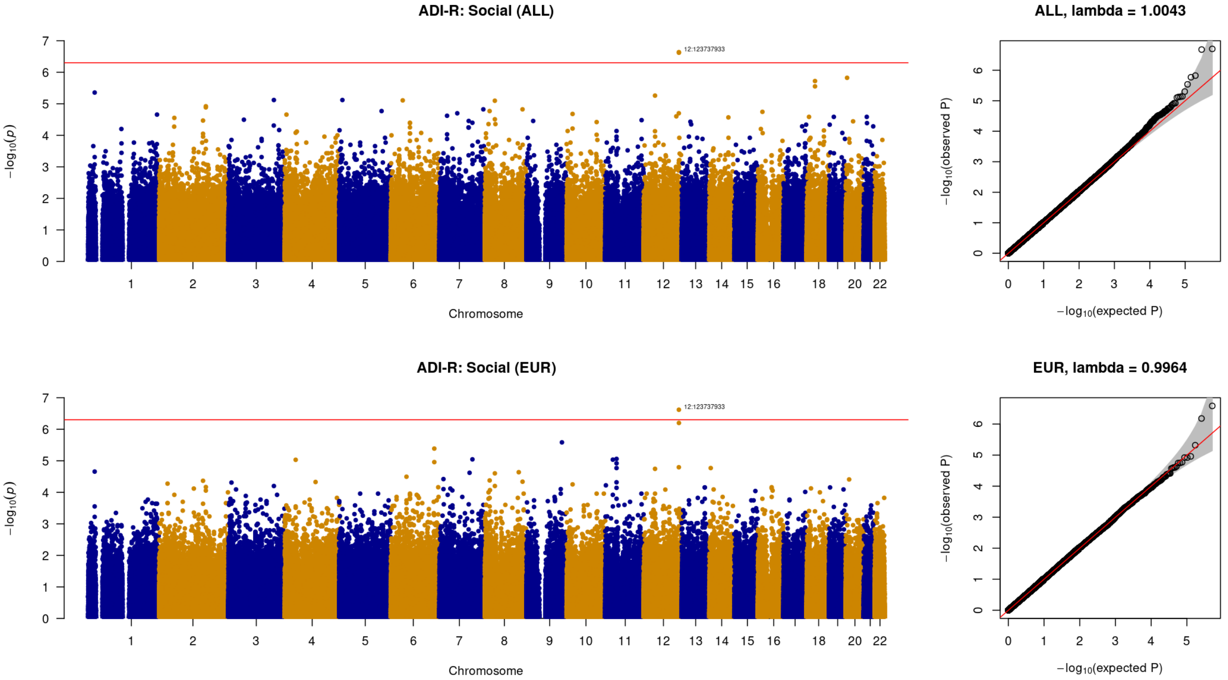

Supplementary Figure 2. Manhattan plots for ADOS Module 1 Play score. In Manhattan plots, the horizontal lines represent P-value threshold of 5×10^{-7}. The variants with top P-value above the threshold in each chromosome are labelled by their genomic loci. The quantile-quantile plots are shown next to the corresponding Manhattan plots. (top) GWAS results using all available individuals. (bottom) Results with European individuals only.

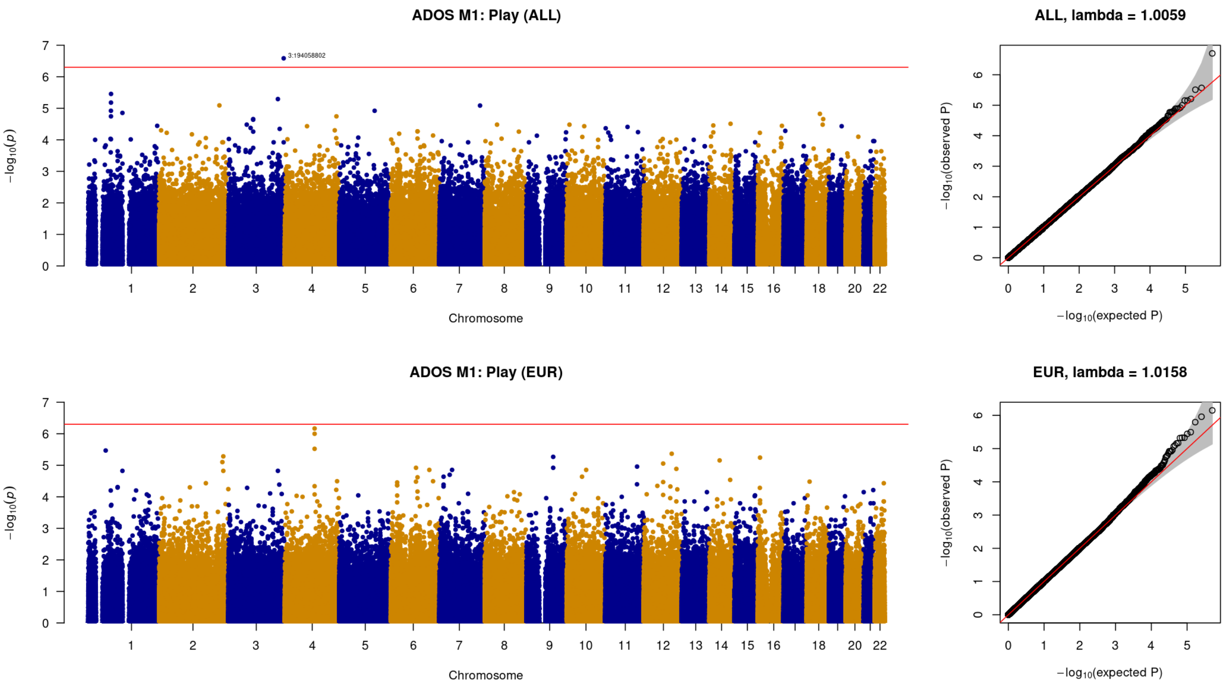

Supplementary Figure 3. Manhattan plots for ADOS Module 3 Communication score. In Manhattan plots, the horizontal lines represent P-value threshold of 5×10^{-7}. The variants with top P-value above the threshold in each chromosome are labelled by their genomic loci. The quantile-quantile plots are shown next to the corresponding Manhattan plots. (top) GWAS results using all available individuals. (bottom) Results with European individuals only.

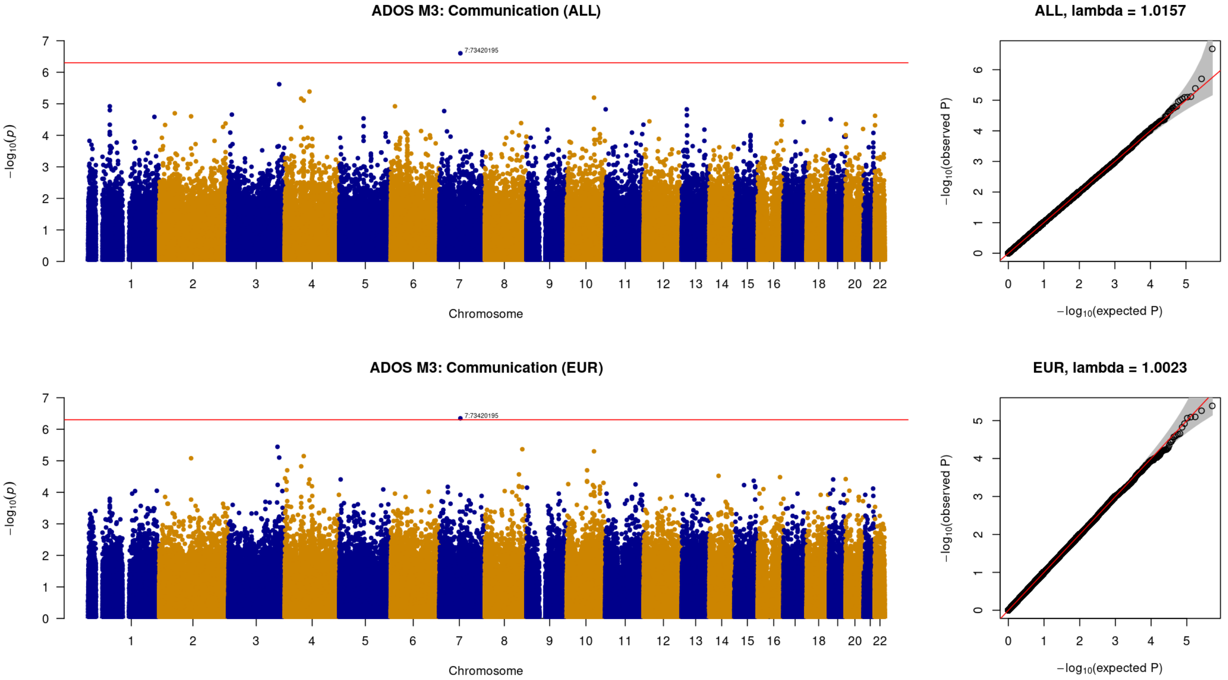

Supplementary Figure 4. Manhattan plots for ADOS Module 3 Stereotyped Behaviors and Restricted Interests score. In Manhattan plots, the horizontal lines represent P-value threshold of 5×10^{-7}. The variants with top P-value above the threshold in each chromosome are labelled by their genomic loci. The quantile-quantile plots are shown next to the corresponding Manhattan plots. (top) GWAS results using all available individuals. (bottom) Results with European individuals only.

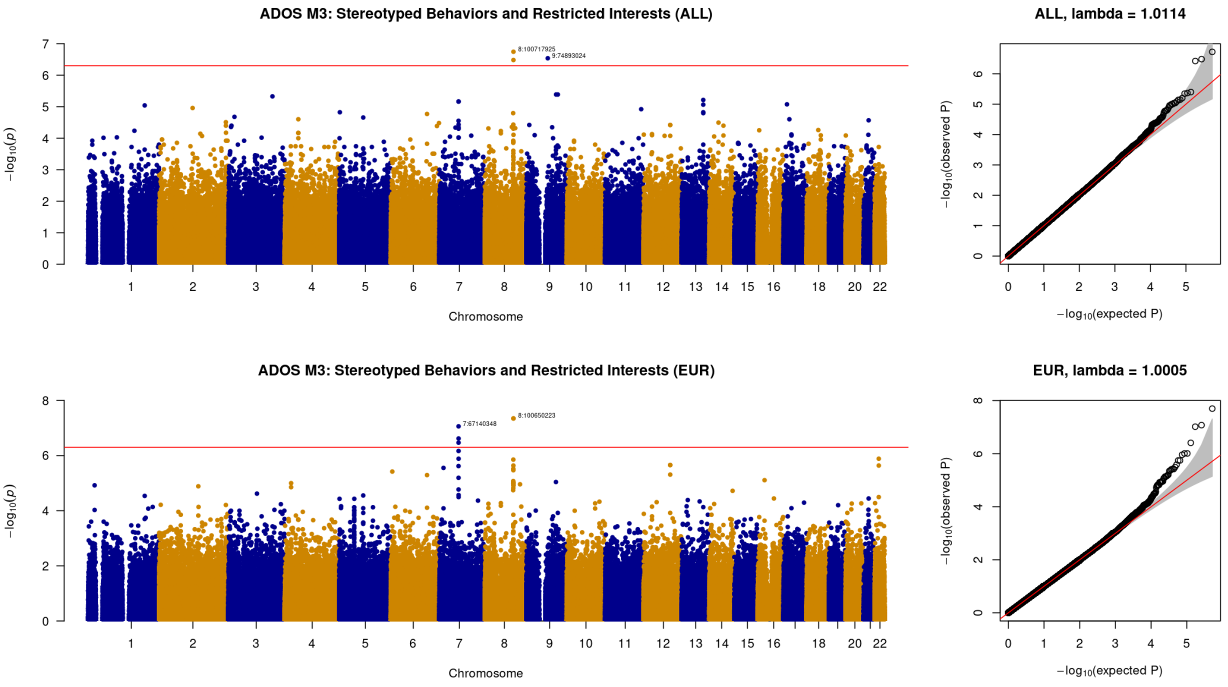

Supplementary Figure 5. Manhattan plots for ADOS Module 3 Total score. In Manhattan plots, the horizontal lines represent P-value threshold of 5×10^{-7}. The variants with top P-value above the threshold in each chromosome are labelled by their genomic loci. The quantile-quantile plots are shown next to the corresponding Manhattan plots. (top) GWAS results using all available individuals. (bottom) Results with European individuals only.

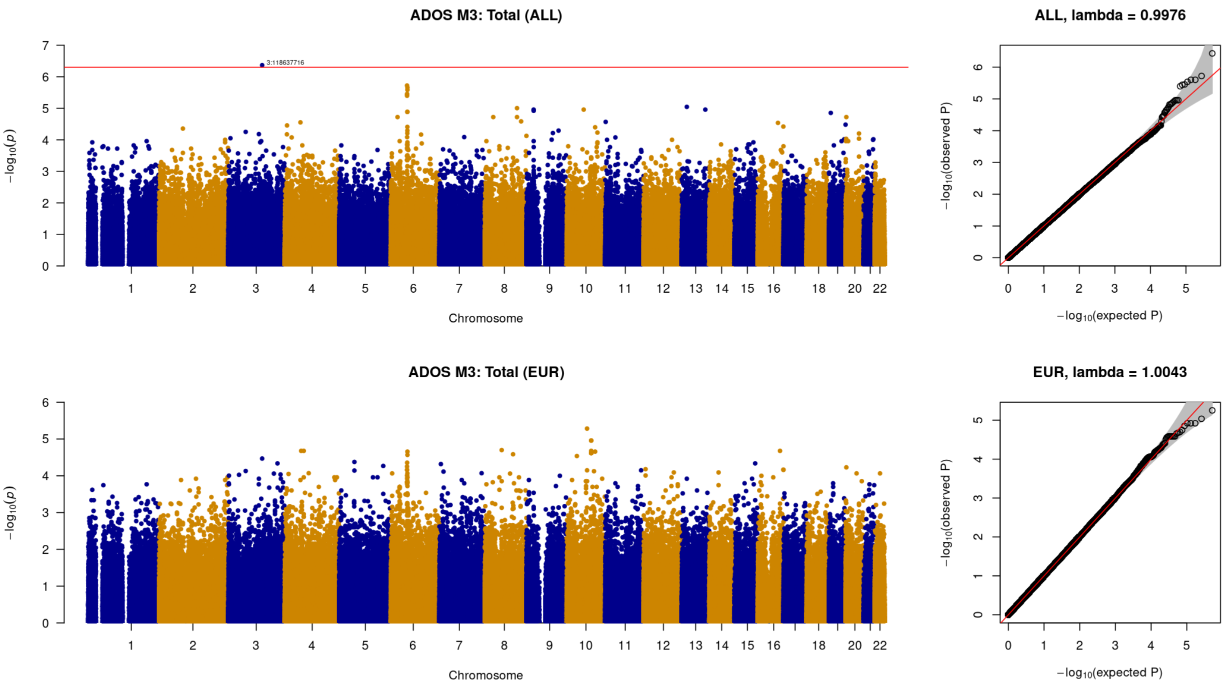

Supplementary Figure 6. Manhattan plots for Raven’s Progressive Colored Matrices (RPCM) score. In Manhattan plots, the horizontal lines represent P-value threshold of 5×10^{-7}. The variants with top P-value above the threshold in each chromosome are labelled by their genomic loci. The quantile-quantile plots are shown next to the corresponding Manhattan plots. (top) GWAS results using all available individuals. (bottom) Results with European individuals only.

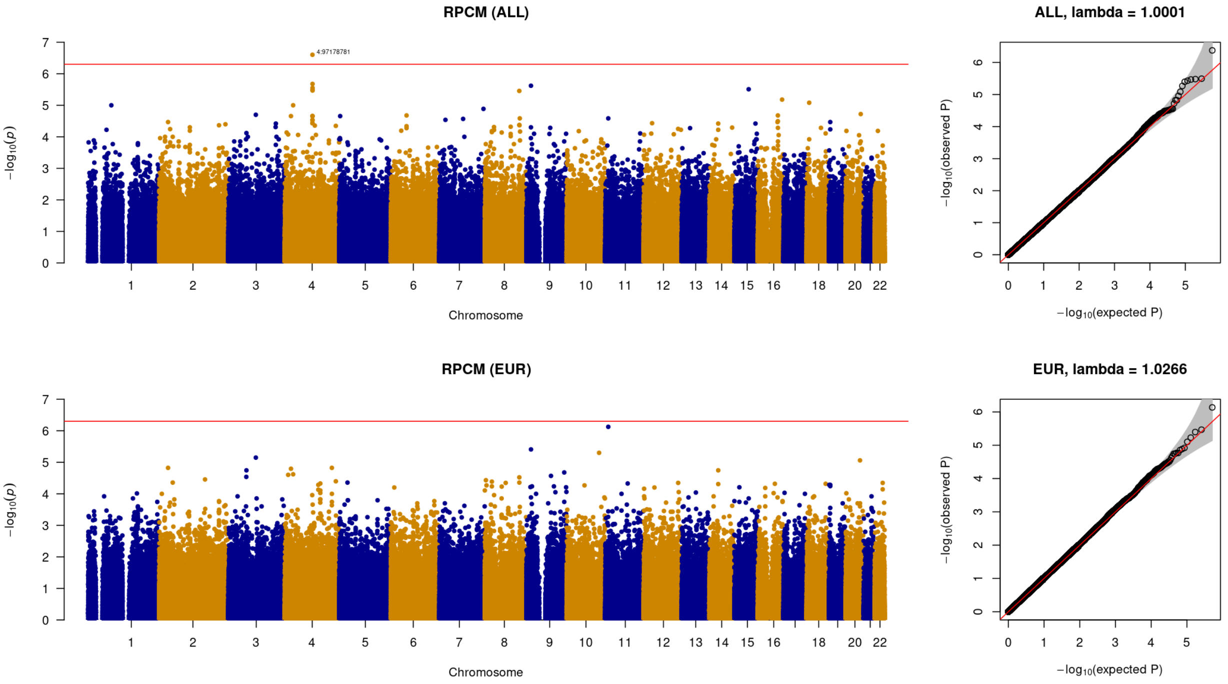

Supplementary Figure 7. Regional plot around the intronic region on *SEMA3E* for ADI-R Communication - Nonverbal Total score. The dashed horizontal lines represent P-value threshold of 5×10^{-6}.

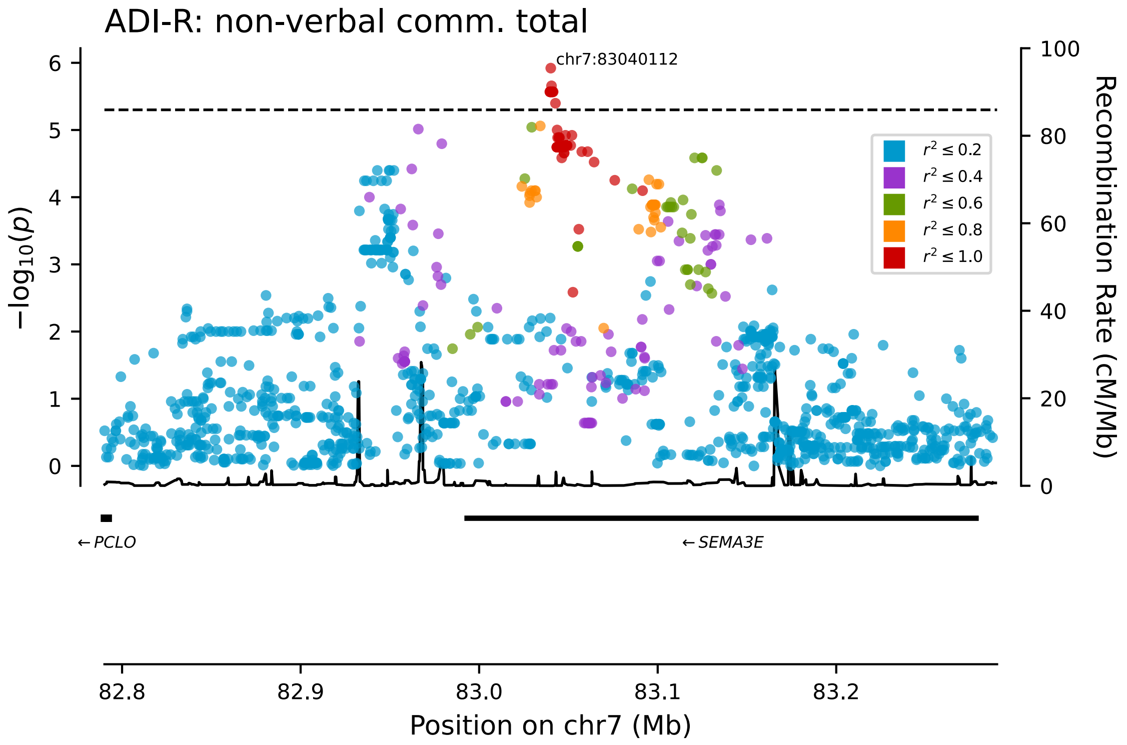

Supplementary Figure 8. Regional plot around the intronic region on *NKAIN3* for maximum head circumference. The dashed horizontal lines represent P-value threshold of 5×10^{-6}.

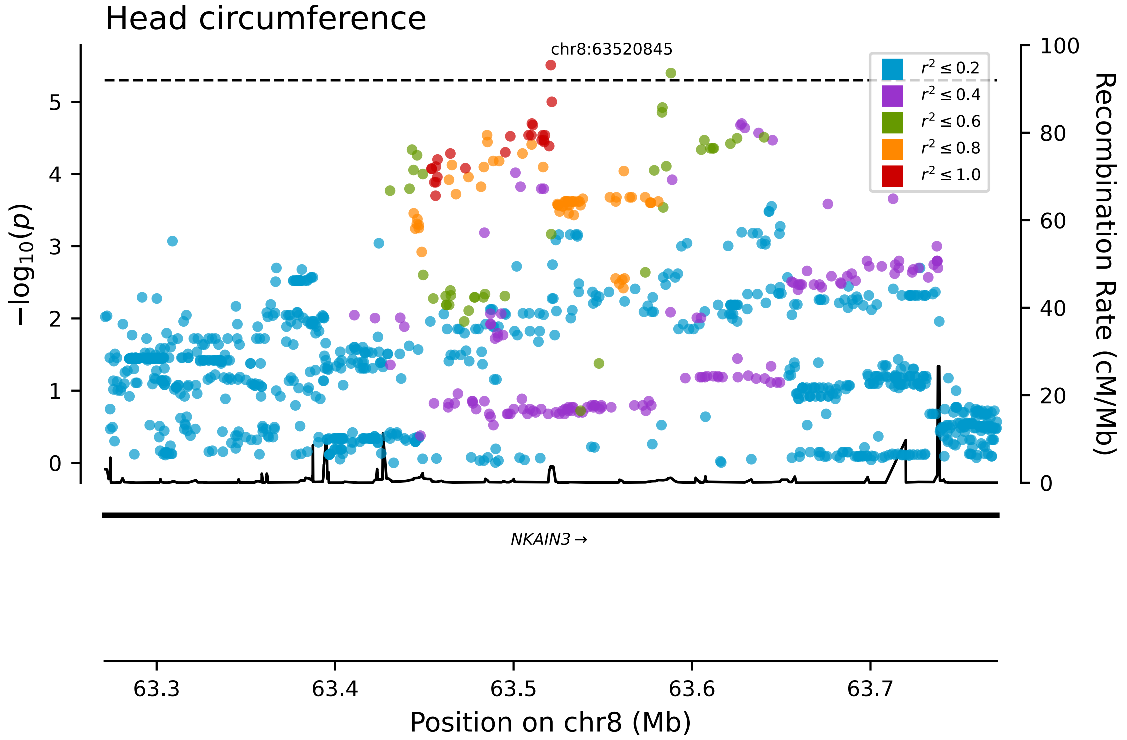

Supplement: Supplementary file 1 — Supplementary Material [file 41398_2022_2179_MOESM1_ESM.pdf]
